# Modeling Trophoblast Cell-Guided Uterine Spiral Artery Transformation in the Rat

**DOI:** 10.3390/ijms23062947

**Published:** 2022-03-09

**Authors:** Vinay Shukla, Michael J. Soares

**Affiliations:** 1 Institute for Reproduction and Perinatal Research, Department of Pathology and Laboratory Medicine, University of Kansas Medical Center, Kansas City, KS 66160, USA; 2 Department of Obstetrics and Gynecology, University of Kansas Medical Center, Kansas City, KS 66160, USA; 3 Center for Perinatal Research, Children’s Mercy Research Institute, Children’s Mercy, Kansas City, MO 64108, USA

**Keywords:** placenta, invasive trophoblast cells, uterine spiral artery remodeling, rat

## Abstract

The rat possesses hemochorial placentation with deep intrauterine trophoblast cell invasion and trophoblast-guided uterine spiral artery remodeling, which resembles human placentation. Uterine spiral arteries are extensively remodeled to deliver sufficient supply of maternal blood and nutrients to the developing fetus. Inadequacies in these key processes negatively impact fetal growth and development. Recent innovations in genome editing combined with effective phenotyping strategies have provided new insights into placental development. Application of these research approaches has highlighted both conserved and species-specific features of hemochorial placentation. The review provides foundational information on rat hemochorial placental development and function during physiological and pathological states, especially as related to the invasive trophoblast cell-guided transformation of uterine spiral arteries. Our goal is to showcase the utility of the rat as a model for in vivo mechanistic investigations targeting regulatory events within the uterine-placental interface.

## 1. Introduction

The placenta is an extraembryonic structure permitting survival of the fetus within the female reproductive tract [1,2]. Among mammals, placentas exhibit differences in their structural organization and vary in their connectivity to their maternal host [3]. Hemochorial placentation is the most invasive form of placentation [3,4]. Trophoblast cells, the specialized cell lineage of the placenta, have a range of functions that ensure the growth and maturation of the fetus [5]. Among these functions is the remodeling of uterine spiral arteries that supply maternal blood to the placenta [5,6,7]. Restructuring uterine spiral arteries at the uterine-placental interface is a multifaceted process involving several cell types. In addition to invasive trophoblast cells, maternal immune cells, including natural killer (NK) cells and macrophages, contribute to uterine spiral artery remodeling [5,8]. In humans, inadequate uterine spiral artery transformation in early pregnancy results in insufficient perfusion of the placenta and fetus and may lead to pregnancy-related disorders such as preeclampsia, fetal growth restriction (FGR), and preterm delivery [9,10]. Mouse, rat, rabbit, human, some non-human primates, and bats are among species possessing a hemochorial placenta [11]. The rat shares deep hemochorial placentation and trophoblast-directed uterine spiral artery restructuring with humans and is an experimentally tractable model [4,12,13].

In this review, we discuss the rat as an experimental model for investigations directed toward elucidation of mechanisms underlying the establishment of the uterine-placental interface. We present a framework for the experimental interrogation of the invasive trophoblast cell lineage and trophoblast-guided uterine spiral artery remodeling. Findings that highlight gene regulatory networks contributing to the formation of the uterine-placental interface of the rat as a model system for studying conserved physiologically relevant mechanisms controlling hemochorial placentation are emphasized in the review. Some data from other model systems are presented to support observations in the rat.

## 2. Organization of the Rat Placentation Site

We will start this section with comments regarding the relationship between maternal tissues and the placenta. The “placenta” is of extraembryonic origin, thus descriptors such as fetal or maternal placenta are not appropriate. Alternatively, the “placentation site” contains both maternal and extraembryonic contributions. Maternal and extraembryonic contributions to the placentation site are essential for a successful pregnancy. Most notably, the maternal uterine stroma transforms into the multi-functional decidua, uterine vasculature is re-engineered to facilitate the flow of nutrients to the conceptus, and maternal immune cells are recruited and/or redistributed within the uterus proximal to the developing placenta [14,15,16]. The placenta is defined by the behavior of its constituent trophoblast cells.

**Figure 1:**
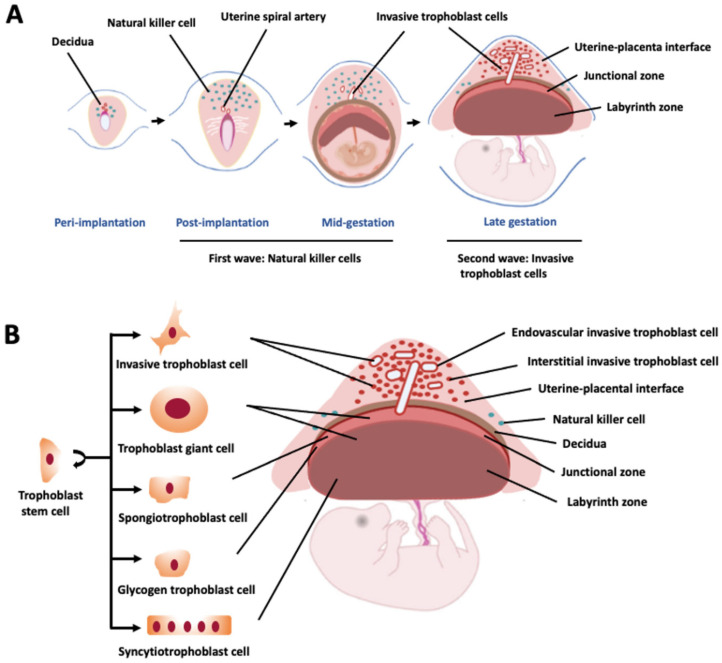
Schematic representations of the rat hemochorial placentation site during gestation. (**A**) Relationship of natural killer (NK) cells and invasive trophoblast cells in transformation of the uterus, including the uterine vasculature. (**B**) Structural compartments and cellular constituents of the mature rat placentation site.

Based on established nomenclature, the rat placenta can be defined based on its histological structure: hemotrichorial, shape: discoid, and invasiveness: deep [11,12,13,17,18,19,20] (Figure 1). The human placenta is similar to the rat placenta in its shape and invasiveness, but its histological structure is distinct and characterized as hemomonochorial. The genesis of the placenta occurs at the initial differentiation event of the embryo when the trophoblast cell lineage is established [21]. Trophectoderm which constitutes the outer cell layer of the blastocyst is the source of trophoblast cell progenitors contributing to the formation of extraembryonic ectoderm and subsequently chorionic ectoderm and the ectoplacental cone [22]. These latter structures are precursors to the two major compartments of the rat placenta known as the labyrinth zone and the junctional zone [23,24]. In the literature, the junctional zone has also been referred to as the basal zone, trophospongiosum, spongiotrophoblast, or spongy region.

Epithelial (trophoblast)-mesenchymal cell interactions dictate placental morphogenesis and formation of the labyrinth zone. The labyrinth zone arises from the interaction of chorionic ectoderm with allantoic mesoderm and is the site of the three trophoblast cell layers comprising the hemotrichorial histological structure defining the rat placenta [24]. Trophoblast progenitor cell populations within the labyrinth zone fuse to form two syncytiotrophoblast layers or alternatively, undergo endoreduplication and differentiate into trophoblast giant cells [24,25,26]. Syncytiotrophoblast layers importantly contribute to the placental barrier, regulating maternal-fetal nutrient and waste exchange [27,28], whereas trophoblast giant cells possess an endocrine function, which notably includes the secretion of placental lactogens [25].

**Figure 2:**
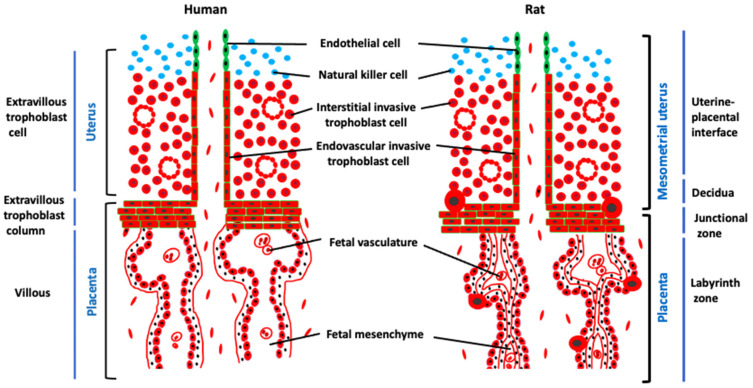
Schematic representations of hemochorial placentation sites of the human and rat highlighting similarities in structural organization. The extravillous trophoblast column and junctional zone are homologous structures of the human and rat, respectively. These structures are the sources of trophoblast cells that invade deep into the uterus. The villous and labyrinth compartments are homologous structures of the human and rat, respectively, and represent the site of maternal-fetal exchange. Histological organization of the villous and labyrinth compartments are defined as hemomonochorial and hemotrichorial, respectively.

The ectoplacental cone gives rise to the junctional zone, a heterogeneous epithelial cell structure containing trophoblast cell-lined channels for blood delivery to the labyrinth zone [23,25,29]. The junctional zone develops between the labyrinth zone and the uterine stroma. Several differentiated cell types arise within the junctional zone and include trophoblast giant cells, spongiotrophoblast cells, glycogen trophoblast cells, and invasive trophoblast cells [5,13]. Progenitor cell-progression to differentiated cell lineages within the junctional zone has not been determined. Cellular constituents of the junctional zone contribute to the maternal endocrine milieu (trophoblast giant cells, spongiotrophoblast cells), placental energy reserve (glycogen trophoblast cells), and to the transformation of the uterus (invasive trophoblast cells) [25,30,31,32]. Invasive trophoblast cells penetrate the mesometrial side of the uterus where uterine spiral arteries/arterioles are situated and extend into a region previously termed the “metrial gland” [33] but now referred to as the “uterine-placental interface” [32]. Invasive trophoblast cells can be categorized by their route of entry into the uterus. Endovascular invasive trophoblast cells enter the vasculature and displace endothelial cells, whereas interstitial invasive trophoblast cells move between blood vessels and directly infiltrate the uterine parenchyma [34,35,36]. In humans, invasive trophoblast cells are referred to as extravillous trophoblast (EVT) cells. Human EVT cells originate from a structure analogous to the junctional zone termed the EVT cell column [5,6,32] (Figure 2). Trophoblast giant cells are polyploid cells formed through a process termed endoreduplication [37,38]. Differentiation of spongiotrophoblast cells, glycogen trophoblast cells, and invasive trophoblast cells are less well understood. A connection between glycogen trophoblast cells and invasive trophoblast cells has been postulated [31] but not yet defined.

Placentation is a dynamic process characterized by significant changes in the structure and function of each placental compartment as gestation advances [13,39,40]. A few examples are noteworthy. Within the junctional zone, glycogen trophoblast cell and invasive trophoblast cell behaviors exhibit prominent temporal changes in numbers and distribution during the last half of pregnancy [13]. Glycogen trophoblast cells first appear after midgestation, exist in clusters, expand in numbers, and then disappear prior to parturition, whereas there is a prominent exit of invasive trophoblast cells from midgestation to term [34,35,36,41]. The appearance of trophoblast giant cells within the labyrinth zone is also gestation stage-dependent and a fixture during the latter days of pregnancy [41].

Many features of rat and mouse placentation are similar; however, prominent differences exist, including demarcation of the junctional and labyrinth compartments and especially the extent of intrauterine trophoblast cell invasion [12,13]. The boundary between placental compartments in the rat is precise resulting in a smooth interface, facilitating routine dissection, which is generally not possible for the mouse [39]. Trophoblast cell invasion in the mouse is shallow and underdeveloped relative to the extensive intrauterine infiltration observed for the rat [34].

## 3. Life Cycle of Invasive Trophoblast Cells

**Figure 3:**
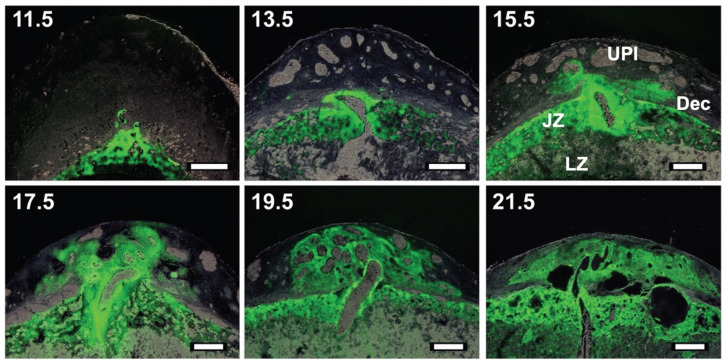
Infiltration of invasive trophoblast cells into the uterine-placental interface during the second half of gestation. Invasive trophoblast cells were identified in placentation sites of wild-type female rats mated with males possessing a chicken β-actin promoter-enhanced green fluorescence protein transgene. Green fluorescence reflects trophoblast cells within the placenta and invasive trophoblast cells which have migrated into the uterine-placental interface. Note the expansion of invasive trophoblast cells into the uterine-placental interface as gestation progresses. Abbreviations: UPI, uterine-placental interface; Dec, decidua; JZ, junctional zone; LZ, labyrinth zone. (Adapted from Reference [46]).

Deep intrauterine trophoblast cell invasion is a characteristic feature of rat placentation [12,13]. Invasive trophoblast cells possess the capacity to recognize, modify, and emulate the behaviors of cells within the uterus. Rat invasive trophoblast cells can be identified by their cytokeratin positive epithelial nature, expression of a unique subset of prolactin family genes and insulin-like growth factor II, accumulation of glycogen, and their position at the uterine site of placental attachment [13,20,34,35,36,42,43,44,45]. Invasive trophoblast cells originate in the junctional zone and migrate from the placenta and into the uterus [12,13] (Figure 3). The first wave of trophoblast cell invasion is restricted to endovascular trophoblast cell colonization of uterine spiral arteries/arterioles, which is initiated at the earliest stages of placentation and is a prominent feature of the midgestation mesometrial decidua [34,35,46]. Endovascular trophoblast cells mimic and supplant endothelial cells [34,35,47,48], destabilize vascular smooth muscle [49], and become embedded in fibrin [12]. A second wave of trophoblast cell invasion begins after gestation day 13.5, as endovascular trophoblast cells penetrate blood vessels beyond the decidua-myometrial boundary and interstitial trophoblast cell invasion is initiated [34,35,36,46]. By the end of pregnancy, interstitial invasive trophoblast cells have infiltrated the entire mesometrial compartment adjacent to the placenta, while some endovascular invasive trophoblast cells become flattened in appearance and embedded in the vascular wall associated with the reappearance of endothelium [34,35,36,46]. Expansion of invasive trophoblast cells within the uterine-placental interface is attributed to their derivation from progenitors located in the junctional zone and not their proliferation within the uterine-placental interface. Evidence exists for both diploid and polyploid invasive trophoblast cells [44,46]. The demise of intrauterine invasive trophoblast cells is signaled by parturition and removal of trophic factors emanating from the placenta [50]. Within a few days of delivery invasive trophoblast cells disappear, an event coupled to macrophage infiltration and restoration of vasomotor control of the uterine vascular smooth muscle [50]. The entire life cycle of rat invasive trophoblast cells transpires within a short interval of time. Invasive trophoblast cells can be tracked from their origin in the junctional zone, dissemination into the uterus, and demise [46,50]. Most importantly, these events exhibit elements of conservation with human placentation and are amenable to laboratory investigation.

## 4. Genetics of Trophoblast Cell Invasion

Strain-specific patterns of placentation are evident in the rat [51,52,53]. The Holtzman Sprague Dawley outbred and ACI, Fischer 344, and Dahl salt-sensitive inbred rat strains exhibit robust deep placentation, characterized by a well-developed junctional zone and widespread intrauterine trophoblast cell invasion [51,52,53]. In contrast, placentation in the Brown Norway rat is more restrained [51]. The most prominent features of the Brown Norway rat placentation site are a growth restricted placenta, including a thin junctional zone, and limited intrauterine trophoblast cell invasion [51,52,53,54]. Attenuated intrauterine trophoblast invasion is associated with retention of NK cells within the late gestation stage uterine-placental interface [51]. Pregnancy outcomes include placental insufficiency that negatively affects fetal growth and survival [51,54]. Brown Norway rat pregnancies are conspicuous for their small litter sizes [51,54]. Genetic influences on placental and fetal size are sexually dimorphic [54]. Brown Norway rat pregnancies exhibit a bias towards more pronounced male placental and female fetal growth restriction [54]. Potential sexual dimorphisms associated with intrauterine trophoblast cell invasion in the Brown Norway rat have not been reported. Chromosome substituted and recombinant inbred rat strains have been used to investigate chromosomal contributions to placental development [53,55]. In addition to structural differences, the Brown Norway rat placenta and especially the Brown Norway rat junctional zone possesses unique transcriptional profiles [53,54]. Among the affected transcripts was an upregulation of prolactin family 5, subfamily a, member 1 (*Prl5a1*) in the Brown Norway rat junctional zone [53]. *Prl5a1* is an invasive trophoblast cell specific transcript, which is not typically expressed in the junctional zone [34,53]. Thus, providing evidence for an aberration in the exodus of invasive trophoblast cells from the Brown Norway rat junctional zone into the uterus. The Brown Norway rat invasive trophoblast cell phenotype is determined by intrinsic properties of trophoblast cells and an ineffectual maternal environment [52,53]. Although, gene regulatory pathways linked to abnormal Brown Norway rat trophoblast cell development have not been reported, insights into inadequacies associated with the Brown Norway rat maternal environment are apparent. Female Brown Norway rats are progesterone resistant and exhibit a compromised decidual response [52]. In humans, poor decidualization is an underlying cause of failed deep placentation resulting in disorders such as preeclampsia [56,57,58].

## 5. Experimental Manipulation of Trophoblast Cell Invasion and Uterine Spiral Artery Remodeling

Trophoblast cell-guided transformation of the uterus is influenced by environmental factors, the cellular constituents of the uterine-placental interface, an assortment of experimental manipulations, and disease states. Trophoblast cell invasion and uterine spiral artery remodeling can be influenced by targeting these structures within the uterus or through actions on the junctional zone and development of the invasive trophoblast cell lineage.

### 5.1. Oxygen Tension

Oxygen is an essential cellular nutrient and a critical regulator of hemochorial placentation [59,60]. Trophoblast cell development occurs in a low oxygen environment [59] and is under the control of hypoxia signaling pathways [60,61,62]. Exposure of pregnant rats to a hypoxic environment beginning after embryo implantation, and including gestation days 8.5 to 9.5, affects placental organization, resulting in an expansion of the junctional zone [46,63] and triggering enhanced development of endovascular invasive trophoblast cells, and their invasion, colonization, and restructuring of uterine spiral arteries [46]. Activation of the invasive trophoblast cell phenotype was guided by a hypoxia inducible factor (HIF) pathway involving HIF1B (also called aryl hydrocarbon nuclear translocator, (ARNT), lysine demethylase 3A (KDM3A), and matrix metallopeptidase 12 (MMP12) [64]. In response to low oxygen tension HIF activates the transcription of a cohort of genes, including KDM3A [47,64]. The trophoblast cell epigenetic landscape is redirected through the demethylating actions of KDM3A on histone 3 lysine 9 (H3K9), leading to further gene activation [64]. Among the genes activated in endovascular invasive trophoblast cells by this pathway is MMP12 [64,65]. Arterial elastin is degraded by MMP12 resulting in restructuring uterine spiral arteries [64,66,67]. The HIF-KDM3A-MMP12 pathway was elucidated using rat trophoblast stem (TS) cells and in vivo genetically manipulated rat models [64,68] and conservation of elements of the pathway determined using human placental tissue specimens and human trophoblast cells [64,65,66,67,69].

The relationship of oxygen and placentation is more complex than simply activating adaptations that facilitate deep placentation. Severe and/or chronic hypoxia and ischemia-reperfusion can lead to placental abnormalities, including disruptions in the junctional zone, impairments in intrauterine trophoblast cell invasion and uterine spiral artery remodeling, intrauterine fetal growth restriction, and failed pregnancy [70,71,72,73,74]. These adverse effects may be mediated via enothelin-1 signaling [70,74] and disruptions in matrix metallopeptidase expression [72]. Adverse consequences of poor oxygenation also emerge from failed placentation and compromised ability of the placenta to adapt to hypoxia [59,75,76]. Enhancing oxygen delivery can rescue some of the unfavorable effects of placental hypoxia [73].

### 5.2. Immune Cells

Two principal immune cell populations (NK cells, macrophages) have been linked to the regulation of trophoblast cell invasion and uterine spiral artery remodeling [77,78,79,80]. The uterine-placental interface exhibits dynamic changes in NK cell abundance, which shows a reciprocal relationship to the presence of invasive trophoblast cells [34,78,81]. NK cells are most prevalent after embryo implantation until midgestation and then diminish in number as gestation advances concomitant with increases in intrauterine trophoblast cell invasion [34,81]. The reciprocal relationship between NK cells and intrauterine trophoblast cell invasion is also demonstrated by the retention of NK cells within the uterine-placental interface in rat pregnancies with compromised intrauterine trophoblast cell invasion [48,51,82,83]. An exception is NK cell expansion in failed conceptus sites [84]. Macrophages are situated throughout the uterine-placental interface, including in locations proximal to uterine spiral arteries [85,86] and exhibit a pronounced increase in number at parturition [87,88,89].

In vivo depletion of NK cells using immunological or genetic approaches is an effective strategy for discerning the biology of NK cells during establishment of the uterine-placental interface [47,90]. Three key observations were made from pregnancies deficient in NK cells [47,90]: (i) NK cells contribute to early events in uterine spiral artery remodeling; (ii) NK cells restrain intrauterine endovascular trophoblast cell invasion; and (iii) placentation sites adapt in the absence of NK cells. NK cells act to disrupt the integrity of the tunica media of uterine arterial vessels decreasing vascular resistance and increasing blood flow to the placenta, engineering the first wave of uterine spiral artery remodeling [47,91]. Endovascular invasive trophoblast cells have similar actions on vascular smooth muscle, engineer a second wave of uterine spiral artery remodeling, and can compensate for the absence of NK cells [34,35,47,90]. The nature of NK cell restraint on endovascular trophoblast cell invasion may be linked to oxygen delivery [47]. NK cells restructure uterine spiral arteries to promote the transfer of oxygenated blood to the developing placenta and their absence leads to hypoxia-mediated adaptations within the placenta, including an expansion of the junctional zone (source of invasive trophoblast cells) and activation of endovascular trophoblast cell invasion into uterine spiral arteries [47,90]. It is also important to appreciate that NK cells are not uniform, they are heterogeneous, and their phenotype can shift from pro-pregnancy to embryotoxic depending on environmental signals and developmental outcomes [92,93,94].

Macrophages possess several roles at the uterine-placental interface [78,79,80]. They contribute to the regulation of angiogenesis, post-implantation uterine tissue repair, uterine spiral artery remodeling, and immune tolerance [79,80]. Most interestingly, they expand in number at term [87,88,89] and have been hypothesized to contribute to post-partum reconstruction of the uterus, including removal of invasive trophoblast cells [50,79,80,95,96]. Macrophages are also linked to pregnancy-dependent uterine-placental interface responses to inflammation and infection [97,98]. Lipopolysaccharide (LPS) exposure is an effective means of inducing an inflammatory response at the uterine-placental interface that is characterized by an increase in macrophage numbers and adverse effects on intrauterine trophoblast cell invasion and uterine spiral artery remodeling [97,99,100,101,102,103]. TLR4 activation and tumor necrosis factor alpha are viewed as drivers of the deleterious actions of LPS on the uterine-placental interface [97,104,105], whereas galectins and interleukin 33 can protect against the unfavorable consequences of LPS exposure [106,107]. Further implication of macrophages as modulators of the uterine-placental interface is achieved through their experimental activation via infusion of adenosine triphosphate (ATP) into pregnant rats [108]. ATP-induced macrophage activation results in restricted interstitial trophoblast cell invasion and impaired uterine spiral artery remodeling [108]. Maternal infection with specific strains of *Porphyromonas gingivalis*, a Gram-negative bacterium, affects the behavior of NK cells and macrophages and negatively impacts trophoblast cell-guided uterine spiral artery remodeling [98].

### 5.3. Drug, Toxicant, and Miscellaneous Exposures

There have been numerous investigations examining the effects of an assortment of drugs and toxicants on the uterine-placental interface with the goal of determining the target tissue mediating negative fetal developmental outcomes [40]. Treatment with chlorpromazine, a phenothiazine used as a tranquilizer, a range of anticancer drugs (cisplatin, 6-mercaptopurine, methyl methanesulfonate, and methotrexate), dexamethasone (glucocorticoid agonist), aryl hydrocarbon receptor agonists (β-naphthoflavone and Arolor 1254), GW501516 (peroxisome proliferator receptor agonist), nicotine, and thyroid hormone dysregulation cause reductions in placental size and structure, including hypoplasia of the junctional zone, abnormalities in junctional zone glycogen trophoblast cell development, and/or decreased intrauterine interstitial trophoblast cell invasion [109,110,111,112,113,114,115,116,117,118,119,120,121,122,123]. A potential relationship of junctional zone development and intrauterine trophoblast cell invasion is logical and informative. However, mechanisms underlying the effects of these various drugs on the uterine-placental interface are poorly understood.

Other agents act directly on the uterine-placental interface impacting the behavior of NK cells, invasive trophoblast cells, and/or uterine spiral artery remodeling. Exposure to tamoxifen, a selective estrogen receptor modulator, has little effect on placental morphogenesis but major disruptive effects on the uterine-placental interface, especially triggering decreases in uterine NK cell accumulation and defective uterine spiral artery transformation at midgestation [124]. In vivo treatment with doxycycline, a tetracycline antibiotic, impairs endovascular trophoblast cell invasion and uterine spiral artery remodeling and placental perfusion [82]. The mode of action of doxycycline may be through inhibition of matrix metallopeptidase activities [82]. 2,3,7,8-Tetrachlorodibenzo-p-dioxin (TCDD) also has profound effects on the uterine-placental interface, but in this case by accelerating endovascular trophoblast cell invasion [125]. Interestingly, in the rat the actions of TCDD are not directly on trophoblast cells [125]. Instead, TCDD signals through the aryl hydrocarbon receptor (AHR) in endothelial cells, which then act to modulate the cellular composition of the uterine-placental interface. β-naphthoflavone similarly targets endothelial cells of uterine spiral arteries but not trophoblast cells [123]. In addition to these conserved actions on endothelial cells, human trophoblast cells respond directly to activators of AHR signaling, a property not characteristic of rat trophoblast cells [125].

Maternal alcohol ingestion during pregnancy can lead to congenital anomalies, including placental and fetal growth restriction [126]. Timing and duration of ethanol exposure differentially affect placentation [127,128]. Chronic maternal ethanol intake during pregnancy in the rat adversely impacts placentation resulting in disruptions in the junctional zone [129,130,131], decreased prevalence of glycogen trophoblast cells, shallow intrauterine trophoblast cell invasion, and failure of uterine spiral artery remodeling [127,129,130]. Some of these aberrations in placentation may be connected to the disruptive effects of maternal ethanol exposure on insulin-like growth factor and NOTCH signaling [129,130]. In contrast, transient maternal ethanol intake around the time of conception leads to an expansion of the junctional zone and glycogen trophoblast cells [128]. The impact of this latter manipulation on intrauterine trophoblast cell invasion and uterine spiral artery remodeling was not reported.

Treatment with ketoconazole (antifungal drug) or a methylhydrazine derivative (anti-cancer drug) resulted in an expansion of the junctional zone, including glycogen trophoblast cell clusters [132,133]. Administration of phthalates, industrial plasticizers, to pregnant rats had contradictory actions [134]. Phthalate treatment increased placental size but led to degenerative changes within the junctional zone. The impact of ketoconazole, methylhydrazine, or phthalates on the invasive trophoblast cell lineage and uterine spiral artery remodeling was not reported.

### 5.4. Disease States

An assortment of disease states affecting pregnancy and placentation can be modeled in the rat. We will provide an overview of research specifically connecting health disorders that affect trophoblast cell-guided uterine spiral artery remodeling.

#### 5.4.1. Diabetes

Diabetes during pregnancy has been simulated in the rat leading to maternal hyperglycemia with consequential effects on placental and fetal development [135]. Placentomegaly is a typical response to maternal hyperglycemia [136,137,138,139,140]. The junctional zone expands in size and glycogen trophoblast cell clusters become more abundant [136,137,138,139], which are characterized by prominent changes in the junctional zone transcriptome [140]. These events within the placentation site are connected to failures in intrauterine trophoblast cell invasion, especially the interstitial invasive trophoblast cell lineage [83,140], and impairments in uterine spiral artery remodeling [141]. Maternal hyperglycemia is also associated with a retention/expansion of NK cells and macrophages within the uterine-placental interface [83] and compromised plasticity of placentation to environmental stressors such as hypoxia [140]. Some of the consequences of maternal hyperglycemia may be mediated by the direct actions of elevated glucose on trophoblast cell development [140]. Mechanisms underlying the effects of maternal hyperglycemia on junctional zone expansion and its potential link to the invasive trophoblast cell lineage, and placental plasticity have not been determined.

#### 5.4.2. Hypertension/Preeclampsia

Placental abnormalities are associated with pregnancy-induced hypertension and preeclampsia and can be the cause or consequence of these pregnancy-related diseases [10,142]. The literature is replete with descriptions of rat “models” of preeclampsia [143]. As a rule, the focus of the reports is generally on elevated maternal blood pressure and kidney dysfunction, which are hallmarks of the symptomology of the human clinical condition known as preeclampsia [143,144]. Prevailing evidence indicates that failures in trophoblast cell-guided uterine spiral artery are the underlying cause of early onset preeclampsia in humans [10,142]. Thus, there could be advantages in modeling these disorders in a species which exhibits deep placentation such as the rat [12,13]. Insults damaging development of the endovascular trophoblast cell lineage and endovascular trophoblast cell-mediated uterine spiral artery remodeling should be at the core of modeling preeclampsia. However, it is rare for research modeling preeclampsia in the rat to investigate biology at the placentation site, especially as the cause of the disorder. It is also important to appreciate a disease process such as preeclampsia is inherently problematic to investigate in a polytocous species such as the rat. Litter size and intrauterine position can influence fetal development [145,146,147,148]. The intrinsic response of a litter-bearing species to an insult that compromises maternal nutrient delivery to the fetus is to sacrifice the health of vulnerable conceptuses allowing the fittest to survive. Experimentally, this becomes a challenge since it is common to observe a range of placental and fetal responses to an insult within the same pregnancy creating an inherent bias for the analysis. In this section the goal is to highlight experimental manipulations causing maternal hypertension or a “preeclampsia-like” condition and their effects on trophoblast cell-guided uterine spiral artery remodeling. In general, these represent the consequences of induction of maternal hypertension or the “preeclampsia-like” condition rather than being the cause of the disorder.

A frequently studied transgenic rat model possessing an activated renin-angiotensin system exhibits maternal hypertension and many of the hallmarks of preeclampsia [149,150,151]. Maternal hypertension arises from mating female rats expressing an angiotensinogen transgene with male rats expressing a human renin transgene [149]. In contrast to predictions based on preeclampsia in humans, this model of preeclampsia displayed enhanced endovascular trophoblast cell invasion and uterine spiral artery remodeling [152,153,154]. Differences in endovascular trophoblast cell invasion were not as pronounced by the end of gestation [153]. The findings could be interpreted to reflect plasticity, a healthy placentation site, and a predictable response to poor perfusion and deprived oxygen delivery during critical early stages of placental morphogenesis [46]. Interestingly, target tissue access to angiotensin II is relevant to the biological outcome observed with this transgenic model. Uteroplacental angiotensin II upregulation correlates with endovascular trophoblast cell invasion and uterine spiral artery remodeling, whereas systemic angiotensin II delivery inhibits trophoblast cell invasion and vascular remodeling [154]. NK cells provide a protective role in this maternal hypertension model [155]. Immune depletion of NK cells after the initiation of intrauterine trophoblast cell invasion (gestation day 15) specifically inhibited interstitial trophoblast cell invasion, but not endovascular trophoblast cell invasion, and resulted in uterine vasculopathy [155].

The stroke prone spontaneous hypertensive rat (SHRSP) has also been used as a model for pregnancy research. The SHRSP model was established through phenotypic selection and inbreeding [156]. Unlike the transgenic model described above, hypertension in the SHRSP rat is not restricted to pregnancy [157]. SHRSP pregnancies are characterized by small litter sizes and placental and fetal growth restriction [157]. Placental growth restriction includes an underdeveloped junctional zone and diminished glycogen trophoblast cells. The SHRSP uterine-placental interface possesses decreased trophoblast cell invasion and vascular remodeling and unlike other examples of shallow trophoblast cell invasion, NK cells do not compensate and expand in number but instead are diminished [157]. The selection process and multigenic phenotype characteristic of the SHRSP model makes analysis a challenge, including the identification of suitable controls for experiments.

A surgical model causing reduced uterine perfusion pressure (RUPP) during the last week of pregnancy has been utilized to model aspects of preeclampsia in the rat [143,158]. As noted by others, the RUPP rat is useful for modeling the consequences of compromised blood delivery to the placenta and thus several aspects of preeclampsia but is not appropriate for investigating a placental etiology for the disorder [158]. The RUPP procedure leads to an expanded junctional zone [159] and impairments in both intrauterine trophoblast invasion and uterine spiral artery remodeling [72].

Some other attempts to model maternal hypertension/preeclampsia in the rat and examine the consequences on intrauterine trophoblast cell guided uterine spiral artery remodeling include maternal hyperinsulinemia, inhibition of heme oxygenase (HO), and treatment with growth arrest-specific 6 (GAS6) [160,161,162,163,164]. All manipulations resulted in elevated blood pressure and intrauterine growth restriction but caused a range of phenotypes at the uterine-placental interface. Endovascular invasive trophoblast cells were more abundant and located deeper within the uterine parenchyma in pregnancies with maternal hyperinsulinemia [161], whereas HO inhibition led to diminished endovascular trophoblast cell-guided uterine spiral artery remodeling [163]. In contrast, endovascular invasive trophoblast cells were not a target of exogenous GAS6 injections but instead, intrauterine interstitial trophoblast cell invasion was diminished [164]. The range of phenotypic responses to these experimental manipulations reflect the complexity of regulatory events controlling intrauterine trophoblast cell invasion and uterine vascular remodeling.

#### 5.4.3. Malnutrition/Obesity/Hyperthermia

The rat uterine-placental interface has been examined following manipulations of diet and other pathophysiologic challenges. Again, our attention focuses on responses of the junctional zone and events at the uterine-placental interface. Generalized maternal undernutrition, protein restriction, and hyperthermia negatively impacted growth of the junctional zone [165,166,167]. In contrast, maternal iron deficiency resulted in an expansion of the size of the junctional zone [168]. Whether these manipulations affect development of the invasive trophoblast cell lineage and/or trophoblast-guided uterine spiral artery remodeling was not reported. A high fat diet results in a diminished junctional zone [169], an early increase in endovascular and interstitial trophoblast cell invasion and later decreased interstitial trophoblast cell invasion [170].

#### 5.4.4. Overview

To a large extent, the preceding sections on the regulation of invasive trophoblast cell-guided uterine spiral artery remodeling, which are summarized in Table 1, have described potentially interesting phenomenology but provide limited insight into mechanisms regulating the uterine-placental interface.

## 6. A Path to Conserved Mechanisms Controlling Invasive Trophoblast Cell-Guided Uterine Transformation

As detailed above, the rat has limitations as a model for investigating human pregnancy-related diseases. However, the rat can be utilized to investigate specific stages of invasive trophoblast cell lineage development and uterine spiral artery remodeling [12,13]. These stages include: (i) cellular dynamics within the junctional zone, a structure analogous to the EVT cell column of the human placentation site, representing respective sites where the invasive trophoblast cell lineage originates; (ii) differentiation of interstitial and endovascular invasive trophoblast cells, and (iii) actions of invasive trophoblast cells on the uterine parenchyma and vasculature. Initially, candidate conserved gene regulatory networks are identified by gene/protein expression profiling in rat and human placentation. Cellular mechanisms can be interrogated using in vitro rat trophoblast stem cell models, including the Rcho-1 trophoblast stem cell line [171] and blastocyst-derived trophoblast stem cell lines [68] and conservation determined using human trophoblast stem cells [172]. It is important to appreciate that all in vitro analyses are artificial and potentially misleading without complementary in vivo physiological assessments [5]. Invasive trophoblast cells can be effectively tracked in vivo [46,173] and the importance of a gene in a physiological context can be evaluated using lentiviral-mediated trophectoderm gene manipulation [174,175] and global genome edited rat models [48,64,125,176]. Roles for phosphoinositide 3-kinase (PI3K)/AKT/fos-related antigen 1 (FOSL1)/JUNB proto-oncogene (JUNB), HIF/KDM3A/MMP12, achaete-scute family bHLH transcription factor 2 (ASCL2), and tissue factor pathway inhibitor (TFPI) as conserved regulators of the invasive trophoblast cell lineage have been demonstrated using this strategy [47,48,64,176,177,178,179,180] (Table 2). PI3K/AKT signaling regulates FOSL1, which cooperates with JUNB, to form a heterodimer and a functional activator protein 1 (AP-1) transcription factor that facilitates endovascular trophoblast cell development during early stages of placentation [177,178,179,180]. As discussed above, the HIF/KDM3A/MMP12 regulatory circuit contributes to invasive trophoblast cell lineage decisions in response to hypoxia [47,64]. ASCL2, a transcription factor, and TFPI, a regulator of hemostasis, act independently to promote invasive trophoblast cell differentiation [48,176]. Results from the experimentation establish the feasibility of the outlined research strategy and identify key nodes of regulation impacting the invasive trophoblast cell lineage and trophoblast-guided uterine spiral artery remodeling.

## 7. Final Thoughts

The placenta is an organ essential to viviparity [181]. Most importantly, maternal resources are redirected through the actions of the placenta to ensure fetal development. As described above, among mammals this task is accomplished using different strategies [3,11,181]. In species with deep placentation, such as the human and rat, trophoblast cells penetrate deep into the uterus to reengineer maternal vasculature, which supply nutrients to the placenta and for transfer to the fetus [4,6,11]. This is a remarkable undertaking. Trophoblast stem/progenitor cells differentiate into invasive trophoblast cells, become motile and capable of traversing and restructuring the uterus, and modulating uterine cell function [5,6]. These tasks must be navigated within the challenges of a genetically foreign landscape and a potentially volatile maternal environment. Invasive trophoblast cells evolved to facilitate viviparity. At this juncture, our knowledge of invasive trophoblast cells is largely descriptive. Tools are needed, especially tractable in vivo model systems, and the implementation of in vivo imaging platforms to explore trophoblast-guided uterine spiral artery remodeling and its impact on oxygen and nutrient delivery. Questions abound regarding the biology of invasive trophoblast cell transformation of the uterus: (i) How do we identify trophoblast stem/progenitor cells that are destined to be invasive trophoblast cells and where are they located within the placenta? In human placentation, these cells are situated at the base of the EVT cell column [6], but this is not the case for the junctional zone of the rat placenta. (ii) What are the regulatory events controlling the expansion, commitment, and differentiation of trophoblast stem/progenitor cells to the invasive trophoblast cell lineage? (iii) What dictates differentiation to an endovascular invasive trophoblast cell versus an interstitial invasive trophoblast cell? These differentiation outcomes may be positional and determined by the proximal milieu or set-up by the systematic activation or inactivation of specific regulatory pathways. (iv) What mechanisms do endovascular and interstitial invasive trophoblast cells use to exit the placenta and traverse the uterus? Cell guidance systems within a blood vessel are different than present in the decidual/stromal compartment. (v) What is the biological importance of endovascular versus interstitial invasive trophoblast cells? (vi) What is the nature of the invasive trophoblast cell-immune cell dialog? Invasive trophoblast cells and NK cells possess similar uterine targets, but their actions are temporally and physically separated. Differences are apparent regarding the relationships of endovascular versus interstitial invasive trophoblast cells and NK cells and possibly macrophages. Invasive trophoblast cell properties ensuring immune protection are not well understood. (vii) How does the uterus control the life cycle of invasive trophoblast cells? (viii) What regulates the demise of invasive trophoblast cells from the uterus following parturition? The rat has much to offer as an in vivo model for investigating invasive trophoblast cells and their biological functions. It is important to appreciate that not all regulatory pathways controlling the uterine-placental interface will be conserved in the rat and human. Species specific regulatory pathways provide insights into nodes of specialization required to address unique needs for a species. Further development and implementation of tools to examine and manipulate individual cell lineages within the uterine-placental are essential for future progress.

## Figures and Tables

**Table 1:** Experimental manipulations impacting the rat uterine-placental interface.

Experimental Maternal Manipulation	Phenotype of the Uterine-Placental Interface	References
*Oxygen*		
Moderate hypoxia during placental morphogenesis	Expansion of the junctional zone; enhanced endovascular invasive trophoblast restructuring of uterine spiral arteries	[46,63,64]
Severe/chronic hypoxia	Impairments in junctional zone development and trophoblast-guided spiral artery remodeling	[70,71,72,73,74]
*Immune cells*		
NK cells	Remodel uterine arterial vessels; restrain intrauterine endovascular trophoblast cell invasion	[47,90]
Macrophages	Uterine angiogenesis, tissue repair, artery remodeling, immune tolerance, postpartum trophoblast removal	[78,83,85,86,87,88,89]
*Drug, toxicant, and miscellaneous exposure*		
Anti-cancer drugs, dexa-methasone, AHR agonists, GW501516, nicotine	Hypoplasia of the junctional zone, abnormalities in glycogen trophoblast cell development, and/or decreased intrauterine interstitial trophoblast cell invasion	[109,110,111,112,113,114,115,116,117,118,119,120,121,122,123]
Tamoxifen	Disrupts the uterine-placental interface; decreases NK cell numbers; defective uterine spiral artery transformation	[124]
Doxycycline	Impairs endovascular trophoblast cell invasion and uterine spiral artery remodeling and placental perfusion	[82]
Dioxin	Accelerates endovascular trophoblast cell invasion	[125]
Chronic ethanol	Decreased glycogen trophoblast cell numbers; shallow trophoblast invasion; failed uterine spiral artery remodeling	[127,129,130,131]
Ketoconazole, methylhydrazine	Expansion of junctional zone and glycogen trophoblast cell clusters	[132,133]
*Disease states*		
Diabetes: assorted hyperglycemia models	Junctional zone and glycogen trophoblast cell expansion; impaired trophoblast invasion and spiral artery remodeling; retention/expansion of NK cells and macrophages	[136,137,138,139,140,141]
Hypertension/preeclampsia: Angiotensinogen-renin transgenic model	Enhanced endovascular trophoblast cell invasion and uterine spiral artery remodeling	[152,153,154]
Hypertension/preeclampsia: Stroke prone spontaneous hypertensive rat (SHSRP)	Impaired junctional zone and glycogen trophoblast cell development; shallow trophoblast cell invasion; failed uterine spiral artery remodeling	[157]
Hypertension/preeclampsia: Reduced uterine perfusion pressure (RUPP) model	Expanded junctional zone and impairments in intrauterine trophoblast cell invasion and uterine spiral artery remodeling	[72,158,159]
Hypertension/preeclampsia: Maternal hyperinsulinemia	Enhanced endovascular trophoblast cell invasion	[161]
Hypertension/preeclampsia: Hemoxygenase inhibition	Diminished endovascular trophoblast cell-guided uterine spiral artery remodeling	[163]
Hypertension/preeclampsia: GAS6 treatment	Decreased interstitial trophoblast cell invasion	[164]
Malnutrition/protein restriction/hyperthermia	Negative impact on junctional zone development	[165,166,167]
Iron deficiency	Expanded junctional zone	[168]
High fat diet	Diminished junctional zone; early increase in intrauterine trophoblast invasion; ate gestation decline in interstitial trophoblast cell invasion	[169,170]

**Table 2:** Regulatory genes affecting the rat uterine-placental interface.

Genes	Mutation	Phenotype	References
*Fosl1*	Trophoblast-LOF ^a^	Inhibition of endovascular trophoblast cell invasion	[178]
*Kdm3a*	Trophoblast-LOF	Disruption of hypoxia-activated endovascular trophoblast cell invasion	[64]
*Mmp12*	Global-LOF	Disruption of hypoxia-activated endovascular trophoblast cell invasion	[64]
*Ascl2*	Global-LOF	Junctional zone dysgenesis and failed intrauterine trophoblast cell invasion	[176]
*Ahr*	Global-LOF	Maternal AHR regulates TCDD-induced endovascular trophoblast cell invasion	[125]
*Tfpi*	Trophoblast-LOF	Inhibition of endovascular and interstitial trophoblast cell invasion	[48]

^a^ LOF, loss of function.

## Data Availability

Not applicable.
